# Impact of a Physical Exercise and Health Education Program on Metabolic Syndrome and Quality of Life in Postmenopausal Breast Cancer Women Undergoing Adjuvant Treatment with Aromatase Inhibitors

**DOI:** 10.3390/medicina60111893

**Published:** 2024-11-18

**Authors:** Pedro Cespedes, Francisco M. Martínez-Arnau, María Dolores Torregrosa, Omar Cauli, Cristina Buigues

**Affiliations:** 1 Department of Nursing, University of Valencia, 46010 Valencia, Spain; pedro.cespedes@uv.es (P.C.); cristina.buigues@uv.es (C.B.); 2 Department of Physiotherapy, University of Valencia, 46010 Valencia, Spain; francisco.m.martinez@uv.es; 3 Frailty and Cognitive Impairment Research Group (FROG), University of Valencia, 46010 Valencia, Spain; 4 Chair of Healthy, Active and Participative Aging, University of Valencia, 46010 Valencia, Spain; 5 Medical Oncology Department, Doctor Peset University Hospital, 46017 Valencia, Spain; torregrosa_dol@gva.es

**Keywords:** multimodal program, physical exercise, health education, breast cancer, postmenopause, aromatase inhibitors, hormone therapy, metabolic syndrome, metabolic health, quality of life

## Abstract

*Background and Objectives*: Adjuvant treatment with aromatase inhibitors (AIs) in breast cancer (BC) survivors can cause adverse effects such as metabolic syndrome (MS) (insulin resistance, central obesity, atherogenic dyslipidemia, and hypertension) associated with morbidity and premature mortality. We evaluate the effect of a multimodal program based on physical exercise and health education on MS and health-related quality of life (QoL) in postmenopausal women with BC under AIs. *Methods*: A total of 56 postmenopausal women, diagnosed with BC, aged 60 years or older (mean age 67.2 years) and on hormonal treatment with AIs, were included in the multimodal physical exercise and health education program, and evaluated before and after their participation. The assessment of the five criteria of the MS included the following: waist circumference, high blood pressure, fasting glucose, triglycerides, and high-density lipoprotein cholesterol. Two main instruments were used to evaluate the impact of the intervention on QoL: the EORTC QLQ C30 (questionnaire for cancers in general) and the EORTC QLQ BR23 (specifically for breast cancer patients). The EuroQol 5D (EQ-5D) was also used to compare these results. *Results*: The percentage of women meeting the MS criteria was 37.7% at baseline and fell to 15.1% at 3 months after the intervention (*p* = 0.02). The intervention significantly reduced hypertension (*p* < 0.001), central obesity (*p* < 0.001), and the concentration of triglycerides (*p* = 0.016). No significant changes were observed in fasting glucose and HDL concentration. A statistically significant improvement was found in QoL (on both the QLQ30 and BR23 scales). A multivariate regression model analysis identified marital status (being married) (95% CI: 1.728–131.615, *p* = 0.014), and percentage of attendance at health education sessions (95% CI: 1.010–1.211, *p* = 0.029) as positive predictive variables of improvement in MS. *Conclusions*: The implementation of multimodal, community-based programs of physical exercise and health education improve the prevalence of MS and specific criteria of MS and QoL in postmenopausal women with breast cancer receiving AI treatment.

## 1. Introduction

Breast cancer (BC) is the most common malignancy among women both worldwide and in Europe, and the first ranked in both morbidity and mortality [1,2]. Breast cancer represents a heterogeneous group of tumors, both in their clinical behavior and prognosis, but the most frequent subtype of BC, which accounts for approximately 70% of cases, is hormone-dependent breast cancer [3]. One-third of breast cancer cases are found in older women [4], and the majority of these postmenopausal women (around 75%) have the hormone-dependent subtype [3,5]. In adjuvant settings in women with hormone-dependent breast cancer, the first-line pharmacological treatment consists of the administration of aromatase inhibitors (AIs) for 5–10 years with the aim of preventing relapses and increasing their cancer-free survival [3,6]. In postmenopausal women, the main source of estrogen is the conversion of androgens to estrogens in the skin, muscles, and adipose tissue. AIs inhibit the enzyme aromatase (estrogen synthase), a member of the cytochrome P450 monooxygenase superfamily that catalyzes the demethylation of carbon 19 of androgens, producing 18-carbon phenolic estrogens [7].

Although AIs have an established role in increasing survival and reducing relapses [8], they are not exempt from physical and psychological side effects that can affect health-related quality of life (QoL) [9,10,11,12], including menopausal symptoms and osteoarticular pain [13].

AIs also have been shown to be associated with increased blood pressure (BP), dyslipidemia, the development of insulin resistance, and diabetes [14,15,16], especially in postmenopausal women [17]. This association with metabolic factors that make up the so-called metabolic syndrome (MS) increases the risk of cardiovascular events. Boszkiewicz et al., 2022 [10], specifically showed how the impact of AIs on metabolic and cardiovascular health in patients diagnosed with BC increases the risk of cardiovascular events. Modifying healthy habits and lifestyles is therefore especially important in women undergoing AI treatment in order to prevent metabolic and cardiovascular adverse outcomes and further limit BC relapses, since both obesity and hyperinsulinemia associated with insulin resistance are considered risk factors for hormone-dependent tumors such as BC [7,18].

Regular physical exercise, especially if scheduled and supervised, can counteract the physical and psychological adverse effects of AIs, and afford metabolic benefits which also contribute to longer survival and a better QoL [19,20]. By reducing excessive adipose tissue, physical exercise can reduce circulating levels of estrogens and increase sex hormone-binding globulin (SHBG) levels in sedentary and overweight postmenopausal women [21], adding benefit to the anti-estrogen therapy [22]. Other benefits of physical exercise can include the inhibition of sustained tumor proliferation, activation of tumor suppressor genes, activation of apoptosis, activation of the immune system, and the functioning of muscle tissue as a protective endocrine organ due to the secretion of myokines [23,24,25]. 

Furthermore, when associated with good nutritional habits, exercise can help reduce the risk of BC relapses [26,27]. Long-term lifestyle modifications are therefore essential. Improved health education is necessary for these changes in attitudes, lifestyles, and self-care to be sustained, especially among older women. Since physical inactivity is common in this group of women and contributes to morbidity and mortality, health literacy plays a role in motivating people to become or remain physically active [28]. The evidence on the implementation of health education programs seems to support this intervention for improving quality of life in breast cancer survivors [29].

The objective of this study is therefore to analyze whether postmenopausal women with localized BC who receive AI treatment can improve their metabolic health and quality of life through a multimodal program of supervised physical exercise and health education workshops that address healthy lifestyle habits and self-care with AI.

## 2. Materials and Methods

### 2.1. Study Design 

This prospective longitudinal study aims to examine the effects of a physical exercise and health education program on metabolic syndrome and quality of life in postmenopausal women diagnosed with breast cancer who are undergoing adjuvant aromatase inhibitor therapy. The physical evaluations, QoL assessments, and blood analysis were performed in the Medical Oncology department of the Doctor Peset University Hospital (Valencia, Spain). 

### 2.2. Participants and Setting

Women aged 60 years and over referred from the Oncology Service of Doctor Peset University Hospital in Valencia were recruited between September 2023 and April 2024. The oncologist identified the women consecutively as they came in for medical assessments. Women were considered eligible for the program if they had a previous diagnosis of localized hormone-dependent breast cancer, had undergone surgery, and were receiving adjuvant treatment with one of AIs. 

The exclusion criteria were having cognitive impairment or poorly controlled mental illness because it would limit the possibility of understanding the health education sessions and having any physical/functional limitation preventing the possibility of performing the physical exercises included in the program.

This study was performed in a convenience sample. Based on published data about prevalence of metabolic syndrome in Spain in the age group 65–74 years [30], we estimated a priori a prevalence of metabolic syndrome in our study sample of about 40%. The number of women fulfilling the inclusion criteria in the oncology unit was 90. Accepting an alpha risk of 0.05 and a power of 0.8 in a two-tailed test, 46 subjects were needed to detect a statistically significant difference. This calculation was based on an initial proportion of metabolic syndrome of about 50% in the study sample and an expected final proportion of 20% after the intervention.

### 2.3. Intervention and Follow-Up: Multimodal Program

The program consisted of three phases: initial assessment, intervention, and follow-up. The nurse contacted all eligible women by telephone for an initial assessment (Table S1). To adjust the program to the capabilities of the participants, the physiotherapist, a graduate in physical education, a physical exercise and sports specialist, and a nursing professional assessed their functional capacity by performing seven validated physical tests. All the women underwent an initial assessment by the nursing professional on treatment, risk factors, and quality of life. After the program intervention, all the women were invited for a follow-up assessment. This initial assessment was carried out individually in the outpatient clinics of Doctor Peset University Hospital, after informed consent was signed by the nursing staff.

The sociodemographic information, questionnaires, and scales administered, as well as the anthropometric measurements, blood analyses of the patients were carried out by nurses before the program started.

The program, consisting of supervised physical exercise and health education sessions, was undertaken in a senior citizens’ center run by Valencia City Council. The multimodal exercise and health education program consisted of 12 weeks of combined physical training, with two 60 min sessions per week, and six educational workshops on HT and BC-related topics lasting approximately 90 min. The physical exercise branch of the program was supervised by a physical therapist and carried out with the help of an education graduate and a specialist, and was designed by the physical therapist and coordinated by a nurse. The participants were advised to respect their own physical limitations during the training session. The educational workshops were delivered by a nurse (five sessions), an oncologist (one session), and a physical therapist (one session). These sessions sought to empower the women through information provided by professionals on the basic aspects of breast cancer, hormonal treatments, in general, and AIs in particular, and self-care related to all of these. As the sessions were informal, with time for discussion, the women were able to recognize their own risk factors, ask questions about their concerns, and develop strategies for change. The exercise program carried out by the participants consisted of a multicomponent community exercise program supervised by a trained physical therapist [31,32]. The physical exercise consisted of supervised sessions (by a physical therapist) twice a week, and with a purely face-to-face character. Each session consisted of 3 different phases: a first part consisting of a warm-up and joint mobility (5–10 min), then the main part of the session (45–50 min) consisting of sections of 3 sets combining lower and upper limb exercises with elastic bands, followed by a 120–180 s pause with active rest, based on aerobic (balance and/or cardiovascular) exercises, and finally a warm down (3–5 min) with static stretching [23]. The intensity of the exercise was constantly adapted to the capabilities of each participant, taking the effort they perceived in each session individually as a benchmark, and graduated on a scale from 0 (minimum effort) to 10 (maximum effort). Based on this, the objective was to perform 2 weeks of acclimatization (exercise intensity 4–5 out of 10), then 3 weeks at a higher intensity (7 out of 10) and then alternate weeks at maximum intensity (9–10 out of 10) and high intensity (7–8 out of 10).

### 2.4. Data Collection

The assessments carried out before and after the program included the following sections:

#### 2.4.1. Sociodemographic and Clinical Variables

The study variables included sociodemographic characteristics (age, marital status, educational level, employment status and cohabitation, number of daily medications consumed) and clinical variables of breast cancer (time since diagnosis, TNM stage, radiotherapy, chemotherapy, type of surgery, type of AIs, and AI treatment time in months). The type of surgery, stage at diagnosis, and marital status were dichotomized. According to the criteria of [33], medication was classified as polypharmacy, and the most commonly used definition of polypharmacy is taking five or more medications.

#### 2.4.2. Evaluation of the Metabolic Syndrome

The five criteria for metabolic syndrome (abdominal circumference, triglycerides, HDL, systolic and/or diastolic blood pressure, and fasting glucose) were measured according to the third edition of the NCEP-ATP III [34] (cut-off points in Annex 2). The data needed to assess, record, and analyze each of the five MS criteria were obtained using the following tools and measurements: a Dioche anatomical tape measure for measuring Abdominal Perimeter (AP) and an OMRONX3 digital arm Blood Pressure (BP) meter; a blood test that included a complete blood count and a biochemistry test provided the other parameters (fasting blood glucose, triglycerides and HDL). Blood samples were taken in fasting conditions between 7:30 a.m. and 9 a.m. These samples were collected using two vacutainer tubes (one for plasma and the other for serum) containing EDTA, and were measured in the laboratory belonging to the hospital. We used a hemogram to make sure that none of the women had anemia. A Tanita bioelectrical impedance scale (InnerScan BC545N, Tokyo, Japan), was used to measure the participants’ other anthropometric data. The same instruments were used for the measurements of all the women, both before and after the program.

According to the NCEP-ATP III definitions [34], the presence of metabolic syndrome was assessed if they met at least 3 of the 5 criteria (Table S2).

In addition, in order to evaluate which clinical and sociodemographic variable were associated with an improvement in MS we dichotomized MS into two categories: women whose MS improved (i.e., less metabolic syndrome criteria after the intervention compared to baseline), compared to women whose metabolic syndrome criteria were the same or were higher after the intervention compared to the baseline.

#### 2.4.3. Evaluation of Health-Related Quality of Life

The participants were individually administered version 3.0 of the 30-item Quality of Life Questionnaire of the European Organization for Research and Treatment of Cancer (QLQ-C30) developed by the European Organization for Research and Treatment of Cancer (EORTC) to assess their QoL. The specific module created by the same EORTC for BC patients, the QLQ-BR23, and the EuroQol 5D (EQ-5D) were also used. The Spanish versions of the three scales, adapted to our geographic area with prior authorization, were used.

The QLQ30 scale comprises five functional scales, nine symptom scales or items, and a global health status scale. All the scale/single item measures are scored from 0 to 100. A high score on the health status scale/functional and global quality of life represents a high or healthy level of functioning and a high quality of life, while a high score for a symptom scale represents a high level of symptomatology. In addition, following the proposal of [34], the EORTC QLQ-C30 Total Score was also calculated. This is calculated from the average of 13 of the 15 QLQ-C30 scales (the Global Quality of Life scale and the Financial Impact scale are not included). Before calculating the average, the symptom scales must be inverted to obtain a uniform direction for all the scales. The correct way to operationalize this variable according to the same reference is as follows: Physical Functioning + Role Functioning + Social Functioning + Emotional Functioning + Cognitive_Functioning + (100-Fatigue) + (100-Pain) + (100-Nausea_Vomiting) + (100-Dyspnoea) + (100-Sleeping_Disturbances) + (100-Appetite_Loss) + (100-Constipation) + (100-Diarrhea))/13.

The QoL module specifically for BC patients (QLQ-BR23) has 23 items divided into four functional scales: Body Image Scale (4 items), Sexual Functioning (2 items), Sexual Enjoyment, and Worry about the Future with only 1 item; and four symptom scales: Arm mobility (3 items), Breast symptoms (4 items), Side effects of systemic therapy (7 items), plus an item on concern about hair loss. 

The EQ-5D scale was also used to compare the findings obtained with the QLQ30 and QLQ-BR23. The EQ-5D [35] is a generic instrument for measuring health-related quality of life (HRQoL) that can be administered to both relatively healthy individuals (general population) and used in groups of patients with different pathologies. It consists of five dimensions (Mobility, Self-care, Usual activities, Pain and discomfort, Anxiety and depression), each with five levels of severity that are described by statements appropriate for that dimension. The EQ5D also has a second part, in which the women’s self-perception of their health is scored from 0 to 100.

### 2.5. Statistical Analysis

Descriptive statistical analyses were performed. Means and SDs were used to describe the continuous variables, while frequencies and percentages were used to describe the categorical variables. The Kolmogorov–Smirnov test, histograms and Q–Q plot graphics were used to determine whether the data followed a normal distribution and Levene’s test was used to check homogeneity of variances. The correlation between the quantitative variables was determined using Spearman’s correlation test (for non-normally distributed data) or Pearson’s correlation test (for normally distributed data).

The paired T-means parametric test and the Wilcoxon non-parametric test were used to analyze the differences pre- and post-intervention. The McNemar test was used to analyze the differences in the dichotomous categorical variables, and the marginal homogeneity test was used for the categorical variables in more than two categories.

The non-parametric Kruskal–Wallis test used to determine the differences between the variables with three groups or more.

Logistic regression analyses were used to predict the probabilities of the different social and health factors that could be associated with an improvement in MS after the intervention. The results, expressed as odds ratios (ORs), 95% confidence intervals (95% CIs), and statistical significance (*p*) were estimated for each variable significantly associated with the bivariate analyses. 

All the statistical analyses were performed using the SPSS software package (version 29.0; SPSS, Inc., Chicago, IL, USA).

## 3. Results

### 3.1. Sociodemographic and Clinical Data

A total of 90 patients with BC who met the inclusion criteria selected for this study were proposed for participation in the program during the recruitment phase (September 2023 and April 2024). These participants were grouped into four successive groups over time (between December 2023 and July 2024). Finally, 53 of the 56 participants completed the entire program successfully (3 of them were unable to participate due to family problems unrelated to the program). The mean age of the 56 participants who started the program was 67.16 ± 4.80 years. The participants had been on AI treatment for an average of 28.8 ± 3.65 months and had been diagnosed 32.93 ± 3.19 months previously. As shown in Table 1, over half of the participants were married (*n* = 32 (57.1%) and only 21.4% lived alone. A total of 53.6% met the polypharmacy criteria. With regard to the use of drugs related to metabolic syndrome, 26 women (46.4%) were taking an antihypertensive, 32 (57.1%) a hypolipemic agent, 6 women (10.7%) a drug against hypertriglyceridemia, 6 women (10.7%) an oral antidiabetic (OAD), and 1 was undergoing insulin therapy. As for the types of AIs that the women were taking, the most frequent was Anastrozole, taken by 41 women (73%). A total of 11 were taking (19.6%) Letrozole and 4 (7.1%) Exemestane. A total of 53 women completed the program. The average attendance at the exercise sessions was 89.3% ± 10.0, and at the health education workshops it was 94.3% ± 11.3.

### 3.2. Evaluation of the Metabolic Syndrome and Sociodemographic and Clinical Variables

**Figure 1:**
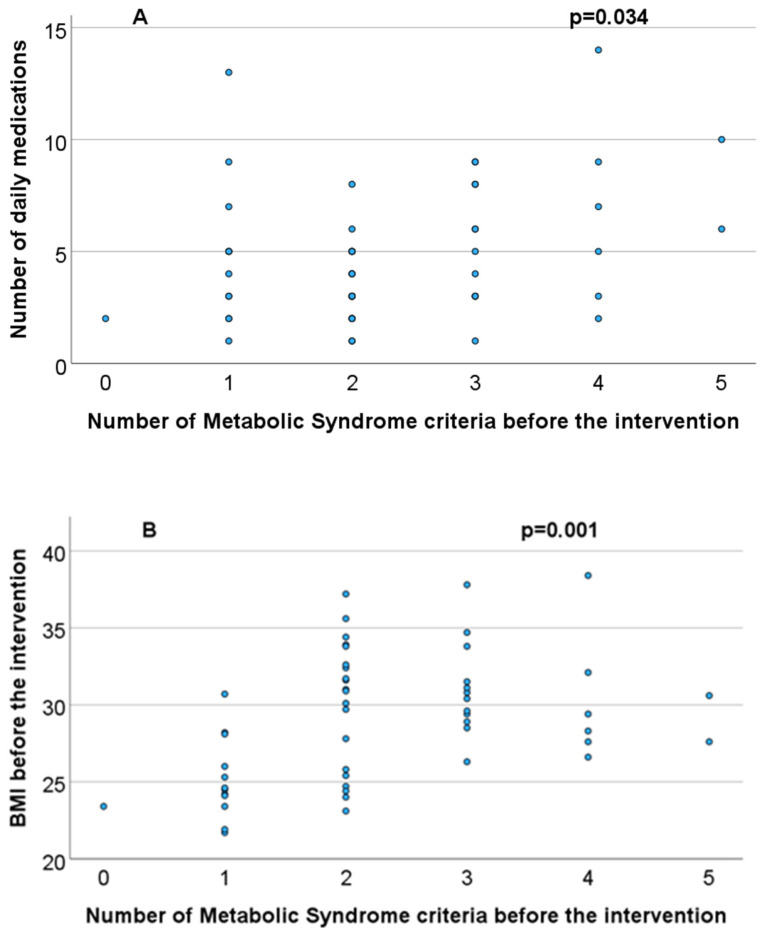
(**A**) Correlation between number of daily medication and number of metabolic syndrome criteria before the intervention. (**B**) Correlation between BMI (Body mass index) before the intervention and number of metabolic syndrome criteria before the intervention.

The number of daily medications that the women took (Rho = 0.292, *p* = 0.034, Spearman correlation test, (Figure 1A), as well as the BMI before the intervention (Rho = 0.439, *p* = 0.01, Spearman correlation test, (Figure 1B), were directly correlated with the number of SD criteria before the intervention. 

There were no statistically significant differences in the age of women between the two groups (*p* = 0.949, Mann–Whitney test) nor any correlation between age and the number of metabolic syndrome criteria that the women met before the program (Rho = −0.090, *p* = 0.520, Spearman correlation test). There were no differences in the metabolic syndrome criteria according to the civil status of the patients (*p* = 0.616, Kruskal–Wallis test). Metabolic syndrome was not associated with the type of AI (*p* = 0.297, Kruskal–Wallis test), nor with the stage at diagnosis (*p* = 0.199, Kruskal–Wallis test), nor having received previous chemotherapy or not (*p* = 0.084, Mann–Whitney) and nor with the dichotomized type of surgery (*p* = 0.500, Mann–Whitney). No significant correlations were found between the number of metabolic syndrome criteria and time since diagnosis (Rho = 0.026, *p* = 0.855, Spearman correlation test). Nor with the length of time the women had been on AI treatment (Rho = −0.103, *p* = 0.462, Spearman correlation test).

### 3.3. Changes in Metabolic Syndrome After the Intervention

There were significant differences in the number of MS criteria met at the end of the program compared to the beginning (*p* < 0.001). Specifically, after the intervention there was a mean reduction of 0.7 MS parameters (2.3 ± 1.1 pre and 1.6 ± 1.1 post). When we analyzed each criterion of metabolic syndrome individually, we found significant differences in the mean of blood pressure (BP) (systolic blood pressure before 143.3 ± 21.9 and systolic blood pressure after 132.3 ± 21.4; *p* < 0.001) and AP (98.47 ± 10.12 before 96 ± 9.31 and after; *p* < 0.001) but not in the means of Triglycerides, Fasting Glucose, and HDL.

**Figure 2:**
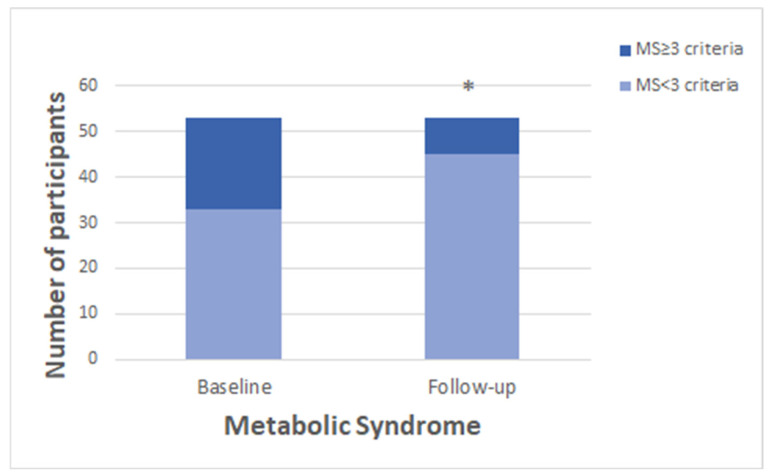
Evaluation of metabolic syndrome based on the presence of the number of criteria after the program compared at the baseline. MS: metabolic syndrome. The presence of metabolic syndrome was assessed if they met at least 3 of the 5 criteria according to the NCEP-ATP III definitions [33] (* significant difference obtained with the McNemar test; *p* = 0.002).

There was a significant difference (*p* = 0.002) in the presence of metabolic syndrome after the program compared to its presence at the baseline (Figure 2).

Similarly, a statistically significant reduction was obtained after participation in the program in the dichotomous individual parameters (with their cut-off points for MS) for BP, AP, and Triglycerides, but not Fasting Glucose or HDL (Table 2).

In addition, after the intervention, there were significant anthropometric changes in BMI (*p* < 0.001), body weight (*p* = 0.008), percentage of muscle mass (*p* = 0.008), percentage of body fat (*p* < 0.001), and the number of participants who met normal weight criteria for their BMI (from 12 (22.6%) to 16 (30.2%); *p* = 0.034). Three of the seven participants who were smokers in the total number of women participants had given up smoking.

### 3.4. Changes in Quality of Life After the Intervention

Significant positive effects on QoL were observed after the intervention in the EORTC QLQ-C30 total score (84.00 ± 12.5 before the program, 90.24 ± 8.73 after the program; *p* = 0.001). Statistically significant positive differences were also found in the global health status (*p* = 0.001) and in all the functional scale scores for QLQ-C30 (Table 3). Negative differences were found for the items related to symptoms after the intervention, for fatigue, nausea, vomiting, pain, dyspnea, constipation, and economic difficulties, and no differences were observed for diarrhea, loss of appetite, and insomnia (Table 3). According to the scoring manual of the EORTC QLQ-C30 questionnaire, a change of 10 points or more is considered a moderate to large clinically significant change [35]. Changes of 10 points or more were observed in both the Global Health Status and the pain subscale (Table 3).

With the specific questionnaire for breast cancer (QLQ-BR23), positive effects were obtained in the functional scale scores body image (*p* < 0.001) and future perspective (*p* = 0.002) and not for sexual functioning and sexual enjoyment (Table 3). A statistically significant reduction in the item scores was found in the four subsections of the symptoms scores (Table 3).

Similarly, significant effects were obtained on the EQ5D scale, in both the sum of the five dimensions (Mobility, Self-care, Usual activities, Pain and discomfort, Anxiety and depression) (*p* < 0.001) and in the self-perception of women’s health scored from 0 to 100 (*p* < 0.001).

### 3.5. Relationship Between Sociodemographic and Clinical Characteristics and the Improvement of Metabolic Syndrome

Logistic regression analysis can be used to evaluate the effects of several factors simultaneously that are likely to be related to the improvement of MS over time, defined as a reduction in the number of MS criteria fulfilled after the program versus the number of MS criteria at baseline. The improvement or otherwise of SD criteria after the intervention was the dependent variable in the logistic regression analyses.

Logistic regression analysis was also used to determine the role of possible confounding factors such as age, dichotomized marital status, percentage of attendance at training and health education sessions, dichotomized chemotherapy treatment, dichotomized type of surgery, number of drugs, BMI, time with AIs, and time since oncological diagnosis QLQ30 total score, QLQ30 Global Health Status and 5Q5D total score pre-program, and improvement or otherwise after the program (Table 4). When selecting the dichotomous variable improvement of MS (decline in the number of criteria) or otherwise (stable/increase in the number of criteria) as the dependent variable, we found significant effects for the dichotomized marital status (95% CI: 1.728–131.615, *p* = 0.014), and the percentage of attendance at health education sessions (95% CI: 1.010–1.211, *p* = 0.029). Other variables showed no significant effect (Table 4).

## 4. Discussion

This study aimed to determine the impact of a multimodal program of combined and supervised physical exercise and health education workshops on the metabolic health and quality of life of breast cancer survivors receiving hormonal treatment with AIs.

The participation rates in the program are important, since patients who have been diagnosed with breast cancer have lower levels of physical exercise, and this applies especially to those with worse functionality, more severe symptoms and less knowledge [36,37,38]. Furthermore, the loss of functionality is greater in older women than in younger patients [39], and the decline in their levels of physical exercise is even more marked.

The program was group-based, since this enhances empathy and social support, which contributes to the development of group cohesion. Mutual support increases their self-efficacy beliefs, and improves their expectations of exercise mastery [40]. This social support also acts as a feedback loop, enhancing the women’s capacity, which can further boost social interactions and self-efficacy, and therefore create a higher level of support received [41]. It was carried out outdoors, in order to reduce anxiety levels and improve well-being during the activity [42].

The intervention was offered as a package and should be viewed as such. Our findings confirmed that the participants benefited from group training, regardless of their age and type of AI.

Physical exercise has multiple benefits for patients diagnosed with BC, and especially on side effects in those receiving HT. Among these benefits, the reduction in the risk of MS is vitally important, due to the bidirectional relationship between MS and BC, especially in postmenopausal women and when the exercise is supervised [43,44]. Our multimodal intervention showed significant and robust effects in this regard. A significant reduction in participants with diagnostic criteria for metabolic syndrome was achieved, in addition to an average reduction in the number of parameters. BP, abdominal circumference, and triglycerides were significantly reduced. BP is one of the most important modifiable risk factors for CVD. The coexistence of hypertension and cancer is common, and its incidence increases with age, and as such diastolic hypertension should be considered a cardiovascular risk factor in these patients [45].

Our results show a significant decline in the BP of the participants after the physical exercise and health education intervention. These results are supported by previous studies of physical activity programs in BC survivors [24,46].

No significant differences were found in HDL. This is consistent with the findings of Travier et al. [47], who evaluated the impact of a 12-week exercise and diet program on metabolic risk in survivors with non-metastatic breast cancer, in which 81.1% of the patients were hormone receptor-positive and 62.2% were postmenopausal. As in our series, significant differences in triglycerides or central obesity were reported in that article. However, unlike our cohort, this study reported differences in fasting glucose and insulin resistance. One possible reason for this is that the article analyzed these variables as continuous, without using cut-off points. The lack of an increase in HDL levels could be due to the duration of the program that would require a longer time to observe a significant effect [47,48,49,50]. Other studies suggested that older men and women may need a different physical training mode to fully benefit and to experience an increase in HDL levels [51], as recently demonstrated by the fact that a 12-week high-intensity interval training increases HDL levels in breast cancer survivors [52]; however, this issue awaits further studies with a longer duration of training as used in our intervention protocol. Regarding the lack of changes in glycaemia, it could be due to the fact that most (86.8% of the study sample) of the women had normal glycaemia before starting the program so it is normal that the day in which the blood sample is taken the glycaemia still is the same after the program. Only seven women (13.2% of the study sample) had type 2 diabetes, but the effect on glycaemia was also non-significant in them when comparing pre- and post-values. It cannot be ruled out that by selecting breast cancer survivors with diabetes, the intervention could lead to a decrease in glycaemia as suggested by international diabetes guidelines regarding physical exercise and glycemic control in people with diabetes [53,54]. However, it should be taken into account that aromatase inhibitors increase insulin resistance [14,55] and this, in turn, may have counteracted the decrease in glycaemia afforded by physical exercise.

The results of logistic regression showed marital status and the percentage of attendance at health education sessions as predictive variables of improvement in the number of MS categories. Among our participants, marital status was equivalent to cohabitation, i.e., there were no women who lived with their partners without being married. This facilitated the coherence of the dichotomization. The married women were more likely to improve in the number of MS categories after the multimodal program than the single, divorced, or widowed women. This association is consistent with previous evidence addressing the relationship between marital status and self-care and self-reported health [56]. In other pathologies, higher levels of adherence to medication are observed in married people, which may influence event-free survival in patients with heart failure [57]. In patients with bone cancer in particular, being married has also been reported to affect patients’ treatment and survival prognosis, thanks to greater material, social, and emotional support, increasing their probability of maintaining healthy lifestyles, especially among middle-aged and older patients [58]. 

The influence of the percentage of attendance at health education sessions reinforces the importance of education in self-care. The patients themselves state that having more information about physical exercise and its usefulness would give them more motivation to do exercise [59]. Health education is therefore crucial in this respect, since informing patients about the side effects of HT and the benefits of physical activity on these effects on the one hand [60], and about healthy habits such as diet, rest, mental health, and pain on the other, can be an effective strategy for increasing adherence to exercise programs and their impact. Suggesting exercise is highly recommended, but there appears to be greater evidence for the usefulness of accompanying the proposal with educational tools and free exercise. These health education workshops also create more time for social interaction.

The fact that we did not observe a significant effect on MS improvement during the time preceding AI therapy suggests that their metabolic syndrome is not caused by AIs, although we cannot rule out a contributory effect. As a result of their lifestyle prior to hormone therapy, some women may already have MS, which may have deteriorated with it or as a result of the life changes associated with the cancer process. We can therefore postulate that MS could be more closely related to ethnicities with strong evidence such as lifestyle, sedentary lifestyle, and dietary habits [61], although we cannot exclude HT, as we have no assessments from prior to the start of HT, or a control group. 

Meanwhile, in line with the benefits obtained after the multimodal program for the women’s QoL, the beneficial impact of physical exercise and health education programs on the QoL of women with BC and in postmenopausal women on AI treatment in particular has been consistently described. Aydin et al. [62] reported an improvement in QoL (also using the QLQ30 questionnaire) and depressive symptoms with combined exercise (aerobics and strength). Wagoner et al. [63] found improvement in fatigue and QoL in women diagnosed with non-metastatic BC using another of the instruments that we use (Euroqol). Pain reduction, improvement in sleep disorders, secretion of endogenous opioids and dopamine, and decreased body fat are some of the numerous ways in which the QoL of this group of patients can improve. This improvement has been evidenced in studies measured with the same instruments we used (QLQ 30, QLQ B23) [64] and in others with some methodological differences, such as the Functional Assessment of Cancer Therapy-Breast (FACT-B), to assess quality of life [24]. Our findings reinforce all these benefits. Regarding the lack of significant effect on the progression of metabolic syndrome on the QoL measured before the program with the different scales, it should be noted that the scores were good, which in addition to the early staging, was due to the fact that the participants had to have completed chemotherapy treatment in order to participate in the program.

Physical exercise also contributes to improving the general health, QoL, mental health and physical function of cancer survivors [23,24,65]. It has been shown to be effective in reducing and preventing adverse effects of breast cancer treatment with HT, such as anxiety, depressive symptoms, fatigue, health-related quality of life, lymphedema (without worsening) and physical function, bone health, and sleep [23]. Physical exercise should be promoted from diagnosis, during treatment, and after completion [66]. Our study also had limitations that should be taken into account. First, all the women in our study came from the same hospital oncology unit which renders it difficult to generalize the results to other countries or cultural contexts. We have no assessments from prior to the start of HT, or comparisons with a simultaneous control group. Furthermore, the fact that we do not know the patients’ lifestyles and physical exercise habits prior to the cancer diagnosis makes it difficult for us to separate the influence of these on the impact of the program. Finally, although it facilitated adherence to the intervention session, the fact that participants were aware of the program’s objectives and the interviews were conducted by researchers may be also considered an important bias. Further studies with larger sample sizes and long-term follow-up are recommended to validate these findings and to assess the sustainability of these improvements over time.

## 5. Conclusions

The implementation of supervised multimodal physical exercise programs and health education in postmenopausal women diagnosed with localized BC on AI treatment can improve their metabolic health and quality of life. Although there may be correlation between metabolic syndrome and quality of life before the program, the improvement in both after an intervention may not be correlated.

## Figures and Tables

**Table 1:** Demographic and clinical characteristics, and physical activity level of all the patients who started the program. The values are numbers (percentages) of patients, unless otherwise indicated.

Demographic Variables	Total Number of Women Who Started the Program (*n* = 56)
Age, years	
Mean (Standard Deviation)	67.16 ± 4.80
Range	60–78
Employment status	
Retired	35 (62.5)
Active	5 (8.9)
Unemployed	16 (28.6)
Marital status	
Married	32 (57.1)
Widowed	9 (16.1)
Divorced	8 (14.3)
Single	7 (12.5)
Medical variables	
BC Staging at diagnosis	
IA	34 (60.7)
IIA	12 (21.4)
IIB	10 (17.9)

**Table 2:** Metabolic syndrome and the different parameters that comprise it of all patients who were able to carry out the program (*n* = 53).

Outcome Variable	Baseline (Prevalence)		12 Weeks (Prevalence)		*p* Value *
Metabolic syndrome	No	Yes	No	Yes	
Fulfilled 3 or more criteria for metabolic syndrome	33	20	45	8	0.002
Analysis of each criterion of metabolic syndrome separately					
Blood pressure	9	44	30	23	<0.001
Systolic blood pressure	11	42	31	22	<0.001
Diastolic blood pressure	17	36	35	18	<0.001
Abdominal perimeter	8	45	18	35	0.002
Fasting glucose	47	6	48	5	1
HDL	38	15	37	16	1
Triglycerides	41	12	48	5	0.016

* obtained with the McNemar test. (HDL: high-density lipoprotein).

**Table 3:** Health related Quality of Life (EORTC QLQ-C30 and QLQ-BR 23) outcome variables and estimated differences in *p* values of all patients who were able to carry out the program (*n* = 53).

Outcome Variable	Baseline: Mean (SD)	12 Weeks: Mean (SD)	*p* Value *
QLQ-30			
Global health status	71.8 ± 15.87	82.1 ± 14.29	0.001
Functional scales			
Physical functioning	87.6 ± 13.98	94.4 ± 8.13	0.001
Role functioning	92.1 ± 17.83	97.5 ± 7.59	0.002
Emotional functioning	78.9 ± 22.76	86.3 ± 15.85	0.002
Cognitive functioning	79.5 ± 21.05	86.1 ± 14.64	<0.001
Social functioning	90.5 ± 19.77	96.6 ± 9.39	0.040
Symptom scales			
Fatigue	21.6 ± 22.20	14 ± 16.33	0.001
Nausea and vomiting	3.5 ± 11.52	0.3 ± 2.34	0.027
Pain	35 ± 27.40	21.4 ± 21.76	0.001
Dyspnea	16.3 ± 25.02	10 ± 16.70	0.011
Insomnia	25.7 ± 33.74	23.2 ± 31.06	0.671
Appetite loss	4.4 ± 13.15	1.9 ± 7.78	0.216
Constipation	22 ± 32	13.2 ± 25.59	0.015
Diarrhea	8.2 ± 20.57	3.8 ± 15.54	0.072
Financial difficulties	8.6 ± 17.69	3.2 ± 13.53	<0.001
QLQ-BR23			
Functional scales			
Body Image	87.9 ± 16.76	92.9 ± 14.91	<0.001
Sexual functioning	85.8 ± 21.52	83.1 ± 21.52	0.412
Sexual enjoyment	83.7 ± 29.68	81.2 ± 28.88	0.636
Future perspective	70.4 ± 31.18	81.2 ± 24.91	0.002
Symptom scales			
Systemic therapy side effects	19.8 ± 13.91	12.4 ± 11.02	<0.001
Breast symptoms	12.5 ± 13.69	7.3 ± 8.74	<0.001
Arm symptoms	16.1 ± 16.98	9.2 ± 13.33	0.002
Upset by hair loss	15.1 ± 28.92	6.9 ± 19.98	0.014

* Obtained with the Wilcoxon test. (SD: standard deviation; QLQ: quality of life questionnaire; BR: breast).

**Table 4:** Logistic regression model: clinical variables associated with the outcome variable (progression or not of metabolic syndrome at follow-up).

Variables	*p* Value	Exp (B)	95% CI	
			LL	UL
Age	0.328	−0.089	0.766	1.093
Marital status	0.014 *	2.713	1.728	131.615
Percentage of training sessions	0.403	−0.046	0.858	1.063
Percentage of health education sessions	0.029 *	0.101	1.010	1.211
BMI	0.363	−0.101	0.727	1.124
Number of drugs	0.063	0.370	0.980	2.139
Smoking	0.244	1.862	0.280	148.032
Time since oncological diagnosis	0.405	0.030	0.960	1.105
Time with AIs	0.244	−0.045	0.885	1.031
Chemotherapy	0.492	−0.659	0.079	3.388
Type of surgery	0.448	0.906	0.238	25.785
Staging	0.200	−1.257	0.042	1.945
QLQ30 total score	0.055	0.101	0.998	1.227
Global Health Status QLQ30	0.730	−0.011	0.929	1.053
Q5QD total score	0.148	−2.954	0.001	2.852

(AIs: aromatase inhibitors; BMI: body mass index; LL: lower limit; UL: upper limit). X2 Hosmer–Lemeshow = 7.377, Sig. = 0.497. “Exp (B)” is the odds ratio, the predicted change in odds for a unit increase in the predictor. The “exp” refers to the exponential value of B. * Indicates statistical significance at *p*-Value < 0.05.

## Data Availability

The data presented in this study are available from the corresponding author on request.

## Supplementary Materials

The following supporting information can be downloaded at: https://www.mdpi.com/article/10.3390/medicina60111893/s1, Table S1: Program Features: information on physical exercise and health education sessions; Table S2: MS cut-off points in women. Source: NCEP-ATP III definitions.
