# Supplementary material for: Impact of a Physical Exercise and Health Education Program on Metabolic Syndrome and Quality of Life in Postmenopausal Breast Cancer Women Undergoing Adjuvant Treatment with Aromatase Inhibitors
Source: Medicina (Kaunas). 2024 Nov 18;60(11):1893. doi: 10.3390/medicina60111893 (PMC11596674; doi:10.3390/medicina60111893)
Supplement: Supplementary file 1 [file medicina-60-01893-s001.zip › medicina-3291984-supplementary.pdf]

medicina

MDPI

Supplementary Materials

Table S1: Program Features: Information on physical exercise and health education sessions; Table S2: MS cut-off points in women. Source: NCEP-ATP III definitions.

Table S1: Program Features: Information on physical exercise and health education sessions

**FITT Formula for physical exercise**	
**Frequency (F)**	Twice a week
**Intensity (I)**	Moderate (60-85% maximum heart rate). Aiming for a gradually increasing perceived effort during the programme
**Type (T)**	Combined: Aerobic+strength+flexibility
**Time (T)**	10 min warm-up and joint mobility + 30-35 minutes work + 10 min warm down.<br>For 12 weeks.
**Contents of the health education sessions**	
**Session 1**	Healthy diet
**Session 2**	Hormone therapy and adverse effects
**Session 3**	Physical exercise
**Session 4**	Pain

**Session 5**	Mental health
**Session 6**	Rest and sleep

Table S2: MS cut-off points in women. Source: NCEP-ATP III definitions

Variables	Cut-off points
Abdominal Perimeter	> 88 cm
Triglycerides	150 mg/dL
HDL	< 50 mg/dL
Blood pressure	Systolic Blood Pressure 130 and/or<br>Dyastolic Blood Pressure 85 mmHg
Fasting Glucose	110 mg/dL
